# Cognitive Reserve proxies can modulate motor and non-motor basal ganglia circuits in early Parkinson’s Disease

**DOI:** 10.1007/s11682-023-00829-8

**Published:** 2023-11-23

**Authors:** Sonia Di Tella, Sara Isernia, Monia Cabinio, Federica Rossetto, Francesca Borgnis, Chiara Pagliari, Marta Cazzoli, Jorge Navarro, Maria Caterina Silveri, Francesca Baglio

**Affiliations:** 1 Department of Psychology, Università Cattolica del Sacro Cuore, Milan, Italy; 2 IRCCS Fondazione Don Carlo Gnocchi ONLUS, Milan, Italy

**Keywords:** Cognitive Reserve, Parkinson’s Disease, Basal ganglia, Magnetic resonance imaging, Prefrontal cortex

## Abstract

Parkinson’s Disease (PD) is hallmarked by dysfunctional circuitry between the basal ganglia and dorsolateral-prefrontal cortex. Recently progress has been made in understanding factors contributing to differential susceptibility to pathology mitigating disease-related cognitive decline. Cognitive reserve, the brain processing resources accumulated throughout life while engaged in mentally stimulating activities, can play an important protective role in cognitive performance. We tested the hypothesis that Cognitive Reserve proxies may exert an impact on the basal ganglia and dorsolateral-prefrontal atrophy in early PD. Forty-five early patients with PD and 20 age-gender-matched healthy controls (HC) completed the Cognitive Reserve Index questionnaire to quantify Cognitive Reserve proxies by three indexes (*CRI-Education, CRI-Working Activity, CRI-Leisure Time*) and a structural MRI examination (3T). Morphometrical indexes for basal ganglia (bilateral putamen, caudate, pallidum volume) and dorsolateral-prefrontal cortex (cortical thickness) were computed. Significant differences between HC and PD were tested by direct comparisons in demographics, cognitive level, and cognitive reserve proxies indexes. Then two multiple regression analyses were performed to identify predictors of the basal ganglia and dorsolateral-prefrontal cortex structural integrity. Regression analysis revealed that basal ganglia volume was significantly predicted by CRI-Education (p_FDR_ = 0.029), sex (p_FDR_ = 0.029), and Total Intracranial Volume (p_FDR_ < 0.001). Instead, the dorsolateral-prefrontal thickness was predicted by CRI-Leisure Time (p_FDR_ = 0.030) and age (p_FDR_ = 0.010). Cognitive Reserve proxies, especially education and leisure-time activities, can play a protective role on the structural integrity of the basal ganglia and dorsolateral-prefrontal cortex, respectively, critical regions hallmarking brain status of early phases of PD.

## Introduction

Parkinson’s Disease (PD), the second most common neurodegenerative disease, is hallmarked by a loss of dopaminergic neurons of the Substantia Nigra (SN) *pars compacta* projecting to the striatum. Non-motor symptoms are also frequently observed in patients with PD. These non-motor symptoms demonstrate phenotypic heterogeneity across disease severity. PD early phases may be characterized by fairly preserved cognitive status not significantly interfering with autonomy in daily living, while severe cognitive complaints may importantly affect daily life independence in advanced disease duration conditions (Goldman & Sieg, 2020). The frontostriatal network circuitry is typically involved in patients with early PD and no clinical evidence of cognitive impairment (Baglio et al., 2011), or restricted to the executive function complaints (Papagno & Trojano, 2018). Indeed, the alteration of basal ganglia (BG) pathways beyond the nigrostriatal system leads to complex motor and non-motor disturbances in these patients, including cognitive deficits principally for dysfunctional circuitry between the striatum and dorsolateral prefrontal cortex (DLPFC) (Williams-Gray et al., 2007).

Recently, understanding reserve-related factors contributing to coping with age-related changes and the “resilience” towards brain pathology is increasingly gaining attention (Stern, 2002; Stern et al., 2020). These factors can account for the mismatch between the severity of pathological changes and the clinical manifestations; they might specifically help understand differential susceptibility to neurodegeneration’s effects, principally on cognition and daily living functioning (Stern et al., 2020).

Cognitive reserve (CR) is a property of the brain that allows for cognitive performance and refers to the processing resources accrued over time due to being engaged in mentally-stimulating activities (Stern et al., 2020, 2023). The involvement in these activities can modify brain anatomy, promoting neurogenesis, angiogenesis, resistance to apoptosis, and neural plasticity (Alvares Pereira et al., 2022). The current evidence suggests that CR can be influenced by multiple genetic and experiential factors, operating at various points or along the lifespan. Among these, the most studied experiential factor associated with CR is education (Arenaza-Urquijo et al., 2017; Bastin et al., 2012; Anatürk et al., 2018; Conti et al., 2021). The working hypothesis is that these CR proxies moderate the relationship between cognitive status and the neurocognitive brain substratum.

In PD, data support that higher levels of education are associated with higher cognitive performance (Ciccarelli et al., 2018, 2022; Guzzetti et al., 2019; Hindle et al., 2014; Loftus et al., 2021). It is, therefore, possible that PD patients with higher CR can cope better with the disease by compensatory mechanisms (Barulli & Stern, 2013). Rouillard and colleagues (Rouillard et al., 2017) emphasize that education and work are important CR proxies showing a protective role on cognition in PD. Thus, higher educational attainment, and a highly mentally stimulating lifestyle, may support cognitive performance in PD and limit cognitive deterioration. Also, these experiential factors may support compensatory mechanisms involving brain areas within and outside the fronto-striatal circuitry in PD (Di Tella et al., 2023).

To the best of our knowledge, to date, no study has investigated the role of CR proxies on the integrity of specific regions of interest in the PD *continuum*. This study aimed to test whether experiential factors could modulate the peculiar BG and DLPFC damage in a cohort of patients with early PD and preserved cognitive status. In fact, neuroimaging studies investigating the disease’s impact on brain morphometry found that patients with early PD show a selective reduction in the BG volume, while in the intermediate stage, progressive cortical atrophy is observed (Li et al., 2022; Sarasso et al., 2021). The extent of structural damage may be predicted by experiential factors from the early stages of the disease, hypothesizing a protective effect of these CR proxies. Especially the non-motor loop connecting DLPFC and the caudate may be considered a good measure of brain status in the early stage of PD when the cognitive level is still preserved (de la Fuente-Fernàndez, 2012; DeLong & Wichmann, 2009; Cools, 2006; Tekin & Cummings, 2002).

## Materials and methods

### Participants

Forty-five early PD patients (mean age (SD): 69.19(7.87) years; 25 males; median disease duration (IQR): 4.00(5.00) years) and 20 age- gender-matched sample of healthy controls (HC) were recruited from the neurological unit of the IRCCS Fondazione Don Carlo Gnocchi ONLUS (Milan, Italy).

Patients’ inclusion criteria were: diagnosis of idiopathic PD according to the Movement Disorder Society Clinical Diagnostic Criteria for PD (Postuma et al., 2015); positive DaTscan; mild-to-moderate disease stage (Modified Hoehn and Yahr, range 1–3) (Goetz et al., 2004; Postuma et al., 2015); stable therapy with either L-Dopa or dopamine agonists; and absence of on-off fluctuations and dyskinesia due to medication.

Exclusion criteria were: clinical signs meeting criteria for other neurological disorders, including possible atypical parkinsonism; secondary or iatrogenic parkinsonism; major psychiatric disorders; contraindications to MRI scanning.

All PD patients underwent a clinical evaluation, including a neurologic examination, and both PD and HC performed a neuropsychological screening, including the Montreal Cognitive Assessment (MoCA) (Table 1).

For the neurological examination of PD patients, MDS-UPDRS Part III, in addition to the total score, four-factor scores were calculated: tremor, bradykinesia of upper and lower limb extremity, rigidity, axial (midline function), accordingly to Li et al. (2018). HC completed a neurological evaluation to rule out neuropsychiatric disorders and systemic and neurological diseases.

This study was conducted in accordance with the principles of the Helsinki Declaration and was approved by the IRCCS Don Carlo Gnocchi Foundation Ethics Committee. Each participant signed a written informed consent.

### Assessment of CR proxies

Participants were administered the Cognitive Reserve Index Questionnaire (CRIq; Nucci et al., 2012), a 20-items standardized instrument to quantify the CR proxies derived from education, professional achievements, and leisure activities. Three indexes (CRI-Education; CRI-Working Activity; CRI-Leisure Time) and a single composite score (Cognitive Reserve Index – CRIq) have been calculated.

The CRI-Education index refers to years of schooling and courses taken. The CRI-Working Activity categorizes professional activities into five levels that differ in terms of the cognitive commitment required as well as the level of responsibility assumed. The CRI-Leisure Time index takes into account leisure and recreational activities. The CRIq total score is expressed on a scale with a mean of 100 and a standard deviation of 15; with more detail, it is considered low level if it is less than or equal to 70, medium-low level if between 70 and 84, medium level if between 85 and 114, medium-high level if between 115 and 130, high level if greater than or equal to 130 (Nucci et al., 2012).

### MRI acquisition and data processing

#### MRI acquisition protocol

All subjects underwent a structural MRI examination on a 3T, Siemens PRISMA scanner equipped with a 64-channel coil, including a fluid-attenuated inversion recovery (FLAIR) (0.4 × 0.4 × 1 mm³, TR/TE: 5000/394 ms, FOV: 256 × 230 mm) sequence; diffusion-weighted echo planar images (EPI) (TR/TE: 3600/92 ms, FOV 210 × 210 mm, 50 diffusion directions b = 1000s/mm^2^, 50 diffusion directions b = 2000s/mm^2^, 5 b = 0s/mm^2^, repeated acquisition in Antero-Posterior and Postero-Anterior directions, voxel 2.0 mm^3^); and a T1-3D magnetization-prepared rapid acquisition with gradient echo (MPRAGE) (0.8 mm^3^, TR/TE: 2300/3.1 ms, FOV: 256 × 240 mm).

#### Substantia nigra MRI assessment

Diffusion-weighted MRI images were processed with FMRIB’s Software Library (FSL, http://www.fmrib.ox.ac.uk/fsl). First of all, the susceptibility-induced off-resonance field was estimated with the topup tool (Andersson et al., 2018). After that, susceptibility-induced geometric distortions, as well as distortions caused by eddy currents and subject movement, were corrected with the eddy tool (Andersson & Sotiropoulos, 2016). The estimate of the diffusion tensor was performed within each voxel with dtifit (Behrens et al., 2003). Then, fractional anisotropy (FA) maps were obtained. Individual FA maps were registered to the Montreal Neurological Institute (MNI) FA template with a nonlinear transformation (Andersson et al., 2018). The diffusion tensor was warped accordingly. Each subject’s mean diffusivity (MD) map in MNI standard space was also derived. In accordance with Bergsland et al. (2021), microstructural integrity values (FA, MD) were then extracted for the SN (*pars reticulata* and *pars compacta*) using probability maps in the MNI space, thresholded at 50% and binarized (Pauli et al., 2018), to confirm the diagnosis of PD.

#### Brain morphometry of BG and DLPFC

**Fig. 1:**
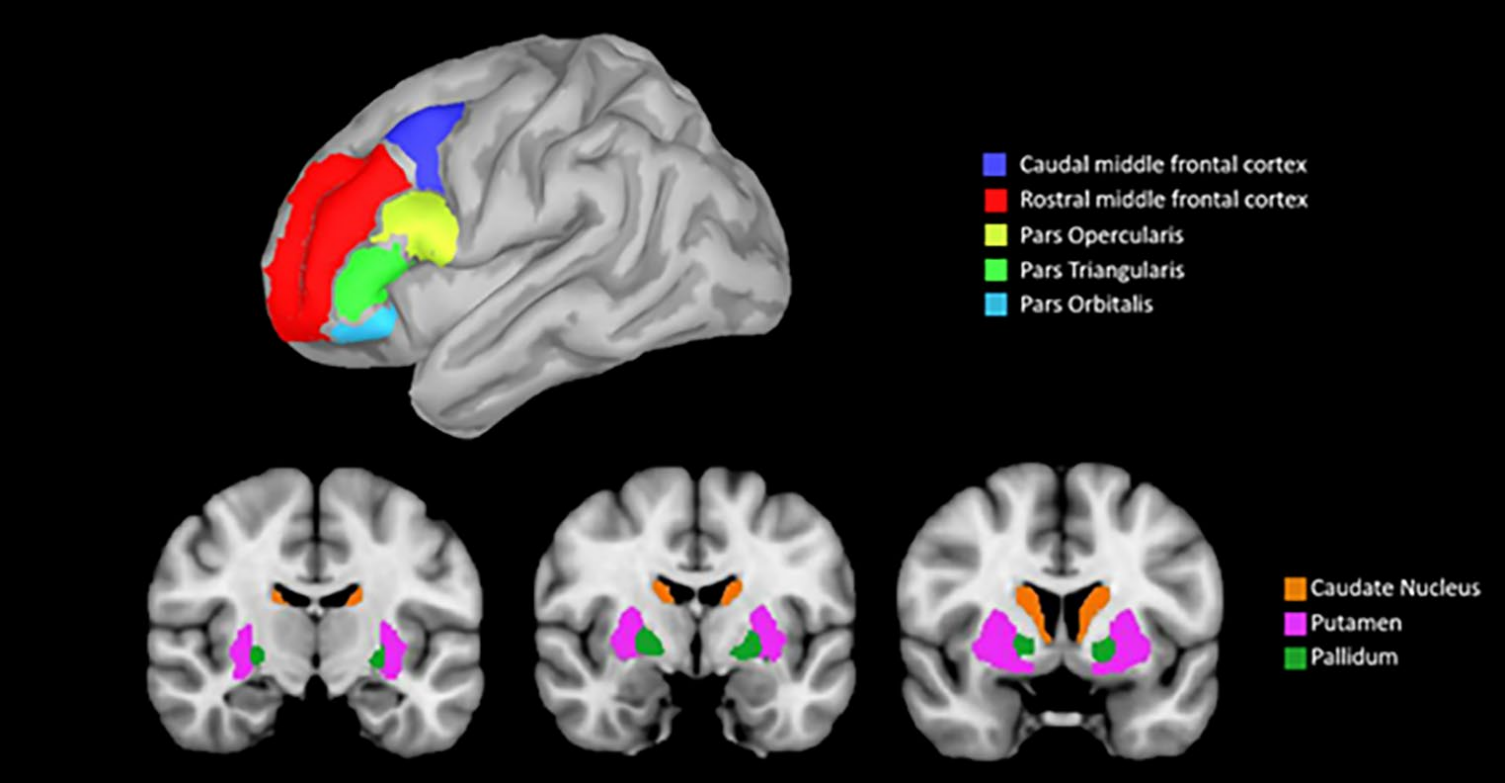
Regions of interest (ROIs) from the Fischl’s et al. (2002) and Desikan’s et al. (2006) atlases

To extract morphometrical data, manual segmentation of white matter (WM) hyperintensities on FLAIR was performed, and T1-3D have been lesion-filled and analyzed using the recon-all pipeline of Freesurfer software (v.6.0, https://surfer.nmr.mgh.harvard.edu/). Parcellation of subcortical and cortical brain regions was performed according to Fischl’s et al. (2002) and Desikan’s et al. (2006) atlases. Manual quality controls were performed according to Klapwijk et al. (2019). Volumetric and cortical thickness measurements of brain areas strongly related to PD pathology were extracted and included in second-level statistics. In particular, we computed BG volume summing up the bilateral putamen, caudate, pallidum subcortical nuclei, and DLPFC summing up bilateral rostral and caudal middle frontal gyrus and inferior frontal gyrus (*pars opercularis, triangularis and orbitalis*). See Fig. 1 for a visual representation of regions of interest (ROIs).

### Statistical analysis

Means and standard deviations or median and interquartile range (IQR) for continuous variables and frequencies for categorical variables were reported. To ensure that the two groups were comparable in terms of socio-demographic characteristics (age, education, gender), cognitive level (MoCA total score), and CRIq indexes (CRIq Education, Working Acitivity, Leisure time and Total score), an independent sample *t*-test (two-tailed)/Chi-squared χ^2^ test as appropriate, was performed.

To evaluate the level of brain neurodegeneration, the MRI morphometric data (BG normalized volumes and DLPFC thickness), the exact Total Intracranial Volume (TIV), and microstructural DTI indexes of SN (FA and MD values of *pars compacta* and *reticulata*) were compared between the two groups using an ANCOVA (age and sex as covariates). The exact Total Intracranial Volume (TIV) was calculated by summing up the total WM, GM, and Cerebrospinal fluid (CSF).

Moreover, BG volumes and DLPFC thickness (dependent variables) were inserted in two separate multiple regression analyses (BG model and DLPFC model) to detect potential predictors of their structural integrity. Correlations (using Pearson’s *r*, Spearman’s *rho* rank correlation, or *r*_*pb*_ point-biserial coefficients as appropriate) were preliminary run to explore the link between morphometrical indexes (BG volumes and DLPFC thickness), sociodemographic (age, sex, and CRIq indexes), clinical variables (H&Y grading and UPDRS scores) and TIV. Moreover, the hierarchical backward approach was adopted in order to simultaneously maximize the predictive power of the final regression and minimize the number of variables included in the model.

Statistical analyses were performed with Jamovi (version 2.3, https://www.jamovi.org/) and SPSS (version 24) softwares. The statistical threshold was set at p < 0.05 two-tailed for all statistical tests, except for correlations (one-tailed). The False Discovery Rate (FDR) type-1 correction was performed to take into account false positive errors (Benjamini & Hochberg, 1995). The magnitude of effects was interpreted as follows: for correlation coefficients, 0.1–0.3 as a small effect; 0.3–0.5 as an intermediate effect; and 0.5 and higher as a strong effect; and for partial η^2^, 0.01–0.05 as a small effect, 0.06–0.13 as a medium effect, and 0.14 and higher as a strong effect coefficient (Cohen, 1988).

## Results

### Clinical characterization of the recruited sample

The baseline comparison revealed that HC and PD groups were matched for age, sex, education, cognitive level, and CRIq indexes (Table 1). In more detail, at the neuropsychological screening, all PD subjects scored above the cut-off of 15.5 (Santangelo et al., 2015), and both groups show medium to high CR (HC = 124.30; PD = 122.98) without significant differences in CRIq indexes (Table 1).

**Table 1:** Description of demographic and clinical characteristics of the PD sample

	HC [N = 20]	PD [N = 45]	Group comparison [*p-value*]
Socio-demographic characteristics			
Age (mean [SD]), years	70.60 [2.95]	69.13 [7.87]	0.423
Education (mean [SD]), years	12.20 [3.41]	11.31 [4.11]	0.401
Gender (Males/Females, n)	7/13	25/20	0.126
MoCA (mean [SD])	24.55 [3.22]	24.09 [3.17]	0.595
Cognitive Reserve Indexes			
CRIq-Education (mean [SD])	110.55 [10.99]	110.58 [13.75]	0.994
CRIq-Working Activity (mean [SD])	114.25 [19.83]	111.09 [25.71]	0.627
CRIq-Leisure Time (mean [SD])	130.10 [24.63]	130.47 [25.99]	0.958
CRIq-Total score (mean [SD])	124.30 [19.72]	122.98 [22.37]	0.821
PD Motor screening			
H & Y (median, [IQR])		2.00 [1.00]	
MDS UPDRS III Total score (mean, [SD])		28.29 [12.55]	
Tremor (mean, [SD])		2.93 [3.47]	
Bradykinesia (mean, [SD])		15.93 [6.81]	
Rigidity (mean, [SD])		5.13 [3.32]	
Axial (mean, [SD])		4.29 [2.78]	
LEDD (mean [SD])		491.20 [273.80]	
Disease duration, years (mean [SD])		4.22 [3.10]	

**Legend.** Independent sample *t*-test was run for group comparison except for the gender variable for which the Chi-squared χ^2^ test was performed. MoCA = Montreal Cognitive Assessment Test; H&Y = Hoehn and Yahr Scale; MDS UPDRS III = Movement Disorder Society Unified Parkinson’s Disease Rating Scale part III; LEDD = Levodopa Equivalent Daily Dose

### Differences between groups on MRI measurements

The ANCOVA results showed (see Table 2): (i) higher MD values in PD than HC in both SN subparts (*pars compacta* p_uncorr_ = 0.005, p_FDR_ = 0.020, partial *η*^*2*^ = 0.13; *pars reticulata *p_uncorr_ = 0.012, p_FDR_ = 0.024, partial *η*^*2*^ = 0.11); (ii) a trend of reduced putamen volumes (p_uncorr_ = 0.039, p_FDR_ = 0.195; partial η² = 0.07) in PD compared to HC; (iii) no differences between the two groups in the thickness of DLPFC.

**Table 2:** Descriptive data and differences between patients and control subjects on MRI measures tested with Analysis of Covariance (ANCOVA), inserting age and sex as covariates

Bilateral ROIs (Mean, [SD])	HC	PD	Group comparison		Effect size [partial *η*^*2*^]
	**[N = 20]**	**[N = 45]**	**[** ***p***_***uncorr***_ **]**	**[** ***p***_***FDR***_ **]**	
**VOLUMES**					
Caudate §	0.59 [0.06]	0.59 [0.06]	0.817	0.817	0.00
Putamen §	0.84 [0.08]	0.81 [0.06]	**0.039**	0.195	0.07
Pallidum §	0.39 [0.04]	0.37 [0.04]	0.156	0.260	0.03
Basal ganglia §	1.82 [0.13]	1.78 [0.12]	0.142	0.260	0.04
TIV §	1042019.52 [112282.02]	1089228.43 [115753.07]	0.677	0.817	0.00
**DIFFUSION PARAMETERS**					
Substantia nigra *pars compacta* FA	0.39 [0.06]	0.38 [0.05]	0.349	0.465	0.02
Substantia nigra *pars compacta* MD#	0.07 [0.01]	0.08 [0.01]	**0.005**	**0.020**	0.13
Substantia nigra *pars reticulata* FA	0.45 [0.05]	0.47 [0.06]	0.572	0.572	0.01
Substantia nigra *pars reticulata* MD#	0.07 [0.01]	0.08 [0.01]	**0.012**	**0.024**	0.11
**THICKNESS**					
Caudal Middle Frontal Gyrus	2.52 [0.10]	2.47 [0.13]	0.065	0.390	0.06
Rostral Middle Frontal Gyrus	2.28 [0.12]	2.23 [0.12]	0.180	0.540	0.03
Pars orbitalis	2.38 [0.14]	2.39 [0.13]	0.854	0.854	0.00
Pars opercularis	2.46 [0.12]	2.45 [0.14]	0.720	0.854	0.00
Pars triangularis	2.31 [0.12]	2.32 [0.12]	0.820	0.854	0.00
DLPFC	2.39 [0.10]	2.37 [0.10]	0.435	0.854	0.01

**Legend.** § = Normalized volumes with TIV (Total Intracranial Volume) multiplied by 100; # = Diffusion values multiplied by 100; FA = fractional anisotropy; MD = mean diffusivity; DLPFC = Dorsolateral Prefrontal Cortex; *p*-values < 0.05 are highlighted in bold; ***p***_***uncorr***_**=** p-values uncorrected; p_FDR_ = p-values corrected for Benjamini & Hochberg False Discovery Rate

### Regression analysis

#### BG model

**Fig. 2:**
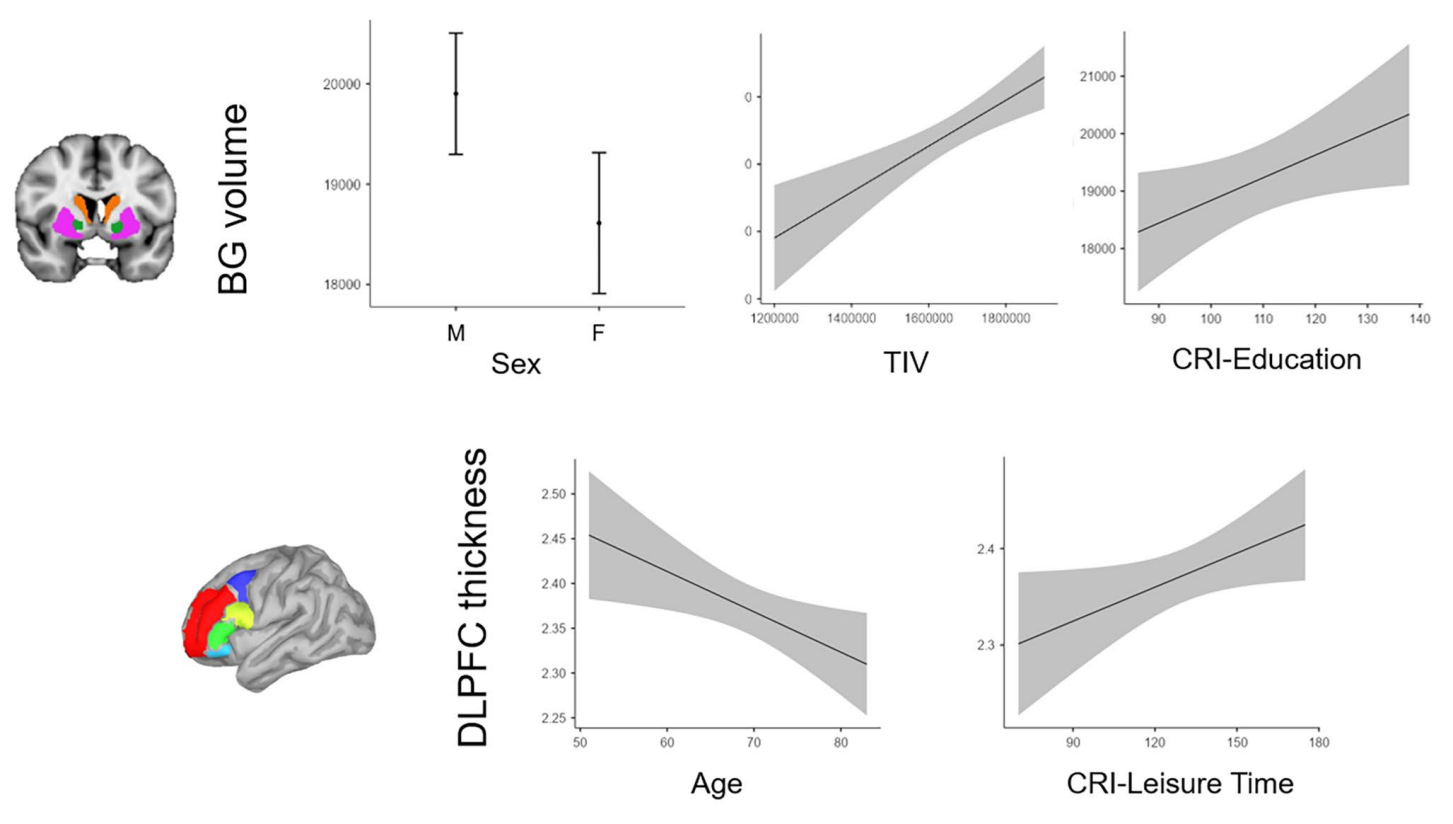
Marginal effects plots of regression analyses on BG volume and DLPFC

Preliminary correlations in the PD group revealed a direct relation between BG volume and TIV (r = 0.809, p < 0.001), CRI- Working Activity (r = 0.307, p = 0.020), and a trend for direct relation with CRI-Education (r = 0.218, p = 0.076); whereas a significant inverse relation emerged with age (r = -0.360, p = 0.015) and a trend for inverse relation with H&Y (*rho *= -0.196, p = 0.098). An association also emerged between BG volume and sex (r_pb_ = -0.661, p < 0.001), with higher volumes associated with the sex of males. The best regression model (R^2^ = 0.734, p < 0.001) revealed that higher BG volume was significantly associated with the sex of males, higher TIV, and higher CRI-Education (Table 3; Fig. 2).

#### DLPFC model

Preliminary correlations in the PD group highlighted a direct relation between DLPFC thickness and CRI-Working Activity (r = 0.345, p = 0.010), CRI-Leisure Time (r = 0.300, p = 0.023) and an inverse relation with age (r = -0.416, p = 0.002) and H&Y (*rho* = -0.216, p = 0.077). The regression analyses (R^2^ = 0.278, p = 0.010; R^2^ = 0.262, p = 0.002) revealed that DLPFC thickness was significantly associated with lower age and higher CRI-Leisure Time (Table 4; Fig. 2).

**Table 3:** Regression analysis on BG volume

BG Model		B	SE	β	t	p	p_FDR_	Omnibus *p*
1	(Intercept)	9730.66	4526.21		2.15	**0.038**	0.076	**< 0.001**
	Age	-39.46	30.60	-0.14	-1.29	0.205	0.305	
	H&Y	-342.15	442.93	-0.07	-0.77	0.445	0.445	
	CRI-Education	45.50	16.92	0.28	2.69	**0.011**	**0.029**	
	CRI-Working Activity	-7.21	9.20	-0.08	-0.78	0.438	0.445	
	CRI-Leisure Time	-9.99	8.16	-0.12	-1.22	0.229	0.305	
	TIV	0.01	0.00	0.57	4.94	**< 0.001**	**< 0.001**	
	Sex (F)	-1372.43	498.08	-0.31	-2.76	**0.009**	**0.029**	
2	(Intercept)	9219.63	4453.78		2.07	**0.045**	0.078	**< 0.001**
	Age	-47.98	28.39	-0.17	-1.69	0.099	0.138	
	CRI-Education	45.76	16.82	0.28	2.72	**0.010**	**0.025**	
	CRI-Working Activity	-6.46	9.10	-0.07	-0.71	0.482	0.482	
	CRI-Leisure Time	-9.14	8.04	-0.11	-1.14	0.263	0.306	
	TIV	0.01	0.00	0.58	5.02	**< 0.001**	**< 0.001**	
	Sex (F)	-1321.93	491.15	-0.30	-2.69	**0.011**	**0.025**	
3	(Intercept)	8296.71	4232.62		1.96	0.057	0.085	**< 0.001**
	Age	-39.51	25.60	-0.14	-1.54	0.131	0.157	
	CRI-Education	40.29	14.86	0.25	2.71	**0.010**	**0.026**	
	CRI-Leisure Time	-9.37	7.98	-0.11	-1.17	0.247	0.247	
	TIV	0.01	0.00	0.58	5.12	**< 0.001**	**< 0.001**	
	Sex (F)	-1234.92	472.58	-0.28	-2.61	**0.013**	**0.026**	
4	(Intercept)	8248.63	4252.45		1.94	0.059	0.073	**< 0.001**
	Age	-36.81	25.62	-0.13	-1.44	0.159	0.159	
	CRI-Education	31.64	12.97	0.19	2.44	**0.019**	**0.031**	
	TIV	0.01	0.00	0.57	5.01	**< 0.001**	**< 0.001**	
	Sex (F)	-1325.59	468.43	-0.30	-2.83	**0.007**	**0.017**	
5	(Intercept)	3863.80	2999.52		1.29	0.205	0.205	**< 0.001**
	CRI-Education	31.26	13.13	0.19	2.38	**0.022**	**0.029**	
	TIV	0.01	0.00	0.65	6.34	**< 0.001**	**< 0.001**	
	Sex (F)	-1137.52	455.57	-0.26	-2.50	**0.017**	**0.029**	

**Legend.** Coeff. = Coefficient; F = females; SE = Standard Error; TIV = Total Intracranial Volume. *p*-values < 0.05 are highlighted in bold. p_FDR_ = p-values corrected for Benjamini & Hochberg False Discovery Rate

**Table 4:** Regression analysis on DLPFC thickness

DLPFC Model		B	SE	β	t	p	*p*_*FDR*_	*omnibus p*
1	(Intercept)	2.43	0.19		12.51	**< 0.001**	**< 0.001**	**0.031**
	Age	-0.01	0.00	-0.40	-2.36	**0.023**	0.080	
	H&Y	0.03	0.04	0.13	0.81	0.423	0.493	
	CRI-Education	0.00	0.00	0.00	0.00	0.999	0.999	
	CRI-Working Activity	0.00	0.00	0.15	0.81	0.423	0.493	
	CRI-Leisure Time	0.00	0.00	0.28	1.73	0.092	0.215	
	Sex (F)	0.03	0.03	0.13	0.92	0.364	0.493	
2	(Intercept)	2.43	0.19		12.91	**< 0.001**	**< 0.001**	**0.015**
	Age	-0.01	0.00	-0.40	-2.47	**0.018**	0.054	
	H&Y	0.03	0.03	0.13	0.82	0.417	0.417	
	CRI-Working Activity	0.00	0.00	0.15	0.92	0.362	0.417	
	CRI-Leisure Time	0.00	0.00	0.28	1.93	0.062	0.124	
	Sex (F)	0.03	0.03	0.13	0.93	0.357	0.417	
3	(Intercept)	2.46	0.19		13.28	**< 0.001**	**< 0.001**	**0.009**
	Age	0.00	0.00	-0.34	-2.36	**0.023**	0.057	
	CRI-Working Activity	0.00	0.00	0.13	0.83	0.411	0.411	
	CRI-Leisure Time	0.00	0.00	0.26	1.82	0.076	0.127	
	Sex (F)	0.02	0.03	0.12	0.84	0.404	0.411	
4	(Intercept)	2.54	0.15		16.95	**< 0.001**	**< 0.001**	**0.005**
	Age	-0.01	0.00	-0.39	-2.86	**0.007**	**0.014**	
	CRI-Leisure Time	0.00	0.00	0.29	2.15	**0.037**	**0.049**	
	Sex (F)	0.02	0.03	0.09	0.64	0.526	0.526	
5	(Intercept)	2.56	0.15		17.47	**< 0.001**	**< 0.001**	**0.002**
	Age	-0.01	0.00	-0.38	-2.85	**0.007**	**0.010**	
	CRI-Leisure Time	0.00	0.00	0.30	2.24	**0.030**	**0.030**	

**Legend.** Coeff. = Coefficient; F = females; SE = Standard Error; TIV = Total Intracranial Volume. *p*-values < 0.05 are highlighted in bold. p_FDR_ = p-values corrected for Benjamini & Hochberg False Discovery Rate

## Discussion

The purpose of this study was to test the hypothesis that the volume of regions mainly involved in the frontostriatal circuit in PD, namely the BG, and DLPFC, was modulated by CR proxies. To this aim, we involved a cohort of early PD patients cognitively fairly preserved.

Our PD cohort presented a similar cognitive level and CRIq indexes compared to an age-sex-education-matched HC group. As expected, the neural profile of patients revealed a microstructural alteration of the SN, initial atrophy in the BG, and preserved integrity in the DLPFC thickness, in line with previous studies including PD participants with the same clinical characteristics of our cohort (low disease duration and severity) (Bergsland et al., 2021; Li et al., 2022; Sarasso et al., 2021).

Considering the role of CR proxies on the integrity of the fronto-striatal circuits, our results supported the study hypothesis in terms of the modulating role of CR proxies on the brain reserve of regions mainly implicated in PD, such as BG and DLPFC. Interestingly, the integrity of BG and DLPFC seemed to be related to differential experiential factors: education exerted protective effects on BG, while leisure time activities significantly modulated DLPFC status.

In detail, concerning subcortical volumes, higher BG volumes were associated with higher CRI-Education, TIV, and male sex. PD patients with more years of education and training courses were more preserved in subcortical regions linked to the initial nigrostriatal dopaminergic denervation. In agreement with Chapko, et al. (Chapko et al., 2018) and Meng & D’Arcy (2012), that consider education as one of the main factors favoring the accumulation of CR proxies, the present research suggests a protective role of education in PD, and, as expected, the volume of these subcortical structures was associated with TIV, which is generally higher in males than females (Ruigrok et al., 2014). Although the role of BG in the control of movement is widely acknowledged, its role has been theorized as linked with other cognitive processes (Phillips & Carr, 1987), such as the acquisition and retention of procedural knowledge. With this in mind, a higher level of study can be linked to these procedural processes and related subcortical neuronal structures. However, to our knowledge, no studies concerning the link between education and the integrity of the BG have been conducted at this time, and further investigations are required.

Moving to the cortical regions involved in the frontostriatal circuit, higher DLPFC thickness was associated with CRI-q leisure time and lower age. Individuals who spent more time in cognitively stimulating leisure and recreational activities, such as reading newspapers, mobile phones use, solving puzzles, multi-day trips, and engaging in other cognitively demanding hobbies, are more preserved in the DLPFC. The positive association between engagement in social-intellectual activities and frontal areas is acknowledged (Arenaza-Urquijo et al., 2017; Bartrés-Faz & Arenaza-Urquijo, 2011). Previous studies demonstrated that CR and structural integrity are linked in older adults’ brains (Anatürk et al., 2018; Conti et al., 2021). Greater GM volumes in individuals with higher CR may correspond to a better tolerance of age-related damage (Bartrés-Faz & Arenaza-Urquijo, 2011; Mortimer et al., 2003; Stern, 2002, 2012), with GM loss concentrated in the prefrontal cortices (Allen et al., 2005; Driscoll et al., 2009; Raz et al., 2004; Schippling et al., 2017; Taki et al., 2013). Similar results were also found in neurodegenerative diseases, reporting an impact of CR, especially in the early stages of Alzheimer’s disease, contributing to the brain maintenance, with lower susceptibility to brain changes associated with the disease (Cohen et al., 2009; Dumurgier et al., 2010; Morbelli et al., 2013; Stern, 2012). Also, pieces of evidence on the effect of lifestyle habits on brain maintenance support a protective role of active social life on changes in cortical thickness, dendritic branching, spine density, neurogenesis, and gliogenesis (Mora, 2013). Our study confirmed this effect in the PD condition, especially in frontal areas, which are traditionally considered to be involved in the planning of complex activities and managing goal-directed behaviors (Badre & Nee, 2018). It has to be mentioned that our PD subjects report a high level of the CRIq-leisure time score. This data may be peculiar to the cohort of this study. In fact, our sample was enrolled in a metropolitan city context, in which cultural- and social-stimulating activities are easily accessible. On the other hand, this result could suggest that leisure time activity is a significant index of reserve in pathological conditions in which the frontostriatal circuit is involved. In fact, pivotal contributions registered the role of environmental enrichment in PD animal models on SN (Klaissle et al., 2012). In fact, an amount of data reveals a great positive effect of housing in new and composite surroundings in mice and rats (Mandolesi et al., 2008; Petrosini et al., 2009).

Concerning the predictive role of age on DLPFC thickness, this association is documented by a large amount of data (Peters et al., 2006). The impact of age on DLPFC is well recognized both in structure and function changes (Cabeza et al., 2018). Age-related neural prefrontal loss is linked with cognitive changes observed with advancing age in which working memory, executive functions, and information processing speed are typically impaired (Anderton, 2002).

Overall, our results suggest that CR proxies and brain neurophysiological status, the brain reserve, are linked through two possible mechanisms. Experiential factors may impact on cognitive performance through brain reserve-related mechanisms. Another possible explanation is the influence of these factors on cognition through mechanisms related to CR (e.g., buffering the association between brain structural integrity and cognitive functioning). Future studies may clarify the direction of this relationship by assessing the cognitive performance together with brain reserve and CR proxies.

### Limitations

We acknowledged that the study presents some limitations: our study has a cross-sectional design. This approach cannot truly disentangle whether the impact of CR proxies may reflect pre-clinical differences in brain structure or differences in the rate of brain loss between PD individuals with different levels of CR. Moreover, the study is focused only on the non-motor brain loop connecting DLPFC and the BG as a marker of brain status in the early stage of PD when the cognitive level is still preserved. Future longitudinal studies are required to evaluate brain maintenance and if CR may mediate the clinical course of PD, mitigating the spread of atrophy in other cortical regions, according to Braak’s staging (Braak et al., 2003) of brain pathology related to PD. Moreover, longitudinal studies are required to evaluate if CR proxies may have a role in predicting the outcome of rehabilitative interventions. However, the strength of this research is that, to date, it is the first study investigating the protective role of CR proxies on BG circuitry in PD. Additionally, CR was considered a multidimensional construct, using a standardized proxy of CR. Future studies may also include additional measures of reserve-related aspects, such as the emerging construct of motor reserve in PD (Sunwoo et al., 2017), introduced as the possibility of the brain being made resilient to neuronal damage by an individual’s engagement in physical exercise throughout their lifespan. Finally, future studies may include an extensive neuropsychological battery to explore executive functioning and how CR proxies mediate the link between frontostriatal circuits status and executive domain behavioral levels.

## Conclusion

Our study supports lower susceptibility to brain burden in PD individuals with higher educational achievement and who spent more time in activities stimulating socio-intellectual abilities before the clinical onset. This suggests that these CR proxy measures may serve as possible markers for the structural integrity of key regions involved in PD from the early stage.

## Data Availability

The data that support the findings of this study are available from the corresponding author upon reasonable request.
